# What Is the Best Approach to Reducing Birth Defects Associated with Isotretinoin?

**DOI:** 10.1371/journal.pmed.0030483

**Published:** 2006-11-21

**Authors:** Lorien Abroms, Edward Maibach, Katherine Lyon-Daniel, Steven R Feldman

## Abstract

Background to the debate: Isotretinoin is an effective treatment for severe acne, a condition which can be physically, emotionally, and socially disabling. Because the drug is teratogenic, causing severe birth defects, women taking the drug are directed to avoid pregnancy. In the United States, a series of risk reduction programs have been implemented that aim to prevent pregnant women from taking the drug and to prevent women taking it from getting pregnant. The most recent, and most stringent, is an Internet-based, performance-linked system called iPLEDGE, which tries to ensure that the drug is dispensed only when there is documentary proof that the patient is not pregnant and is using two forms of birth control. Is iPLEDGE the best way to reduce isotretinoin birth defects, or is it an unproven and overly burdensome system?

## Abstract 2

Isotretinoin is an effective treatment for severe acne, but causes birth defects. Will iPLEDGE, the recently implemented US risk reduction program, reduce isotretinoin birth defects, or is it an unproven and overly burdensome system?

## Lorien Abroms, Edward Maibach, and Katherine Lyon-Daniel's Viewpoint: The Case for Information and Performance-Linked Systems

Untreated acne can be the cause of both significant physical and psychological sequelae [1]. Untreated acne has been associated with difficulties in interpersonal relationships [2], poor employment prospects [3], and suicide [4]. But acne treatment itself can also cause harm.

Isotretinoin—which is effective in the treatment of severe recalcitrant nodular acne [5]—is highly teratogenic. Pregnancies exposed to isotretinoin are at significant risk of serious malformations [6,7]. Since introduction of the drug in 1982, over 2,000 pregnancies have been exposed to isotretinoin, most resulting in spontaneous or elective abortions [8]. Other risks to patients from isotretinoin use include increased risk of depression and suicide, liver damage, and musculoskeletal symptoms [5,9].

Isotretinoin is widely prescribed: each year, over 1.4 million isotretinoin prescriptions are dispensed in the United States [10]. About half of all prescriptions go to females [8], most of whom are in their reproductive years [7]. Many of the prescriptions written for isotretinoin appear to be written “off label”—either as a first-line treatment for severe acne [11] or as treatment for mild to moderate cases of acne [12].

A number of increasingly strenuous risk management programs (RMPs) have been implemented since isotretinoin's introduction. These programs aim to prevent pregnant women from taking the drug and to prevent women taking it from getting pregnant. The newest of these programs, iPLEDGE (Figure 1), became mandatory in the US on March 1, 2006. This program represents the most rigorous risk management program in history for such a widely prescribed drug, involving patients, physicians, pharmacies, and wholesalers [13].

**Figure 1:**
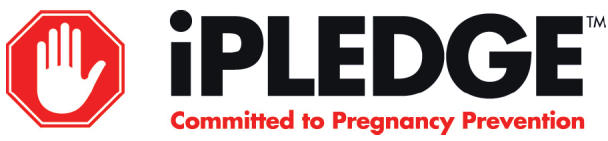
The iPLEDGE Logo iPLEDGE is a “comprehensive program to help you get prepared, plan your treatments, and ensure you don't get pregnant during the course of isotretinoin therapy” (https://www.ipledgeprogram.com). (Figure: Covance Inc.)

In this article we provide a brief history and analysis of isotretinoin RMPs implemented over the past 20 years (summarized in Table 1), which offer general lessons about the effective use of health information and performance requirements. Additionally, we discuss our concerns with the existing iPLEDGE program and provide some suggestions for improving it.

**Table 1:**
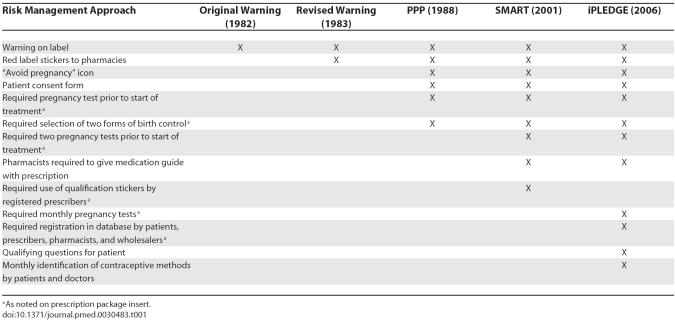
Overview of History of Isotretinoin (Accutane) Risk Management Approaches

The health and risk communication literature and the broader health behavior literature makes clear that provision of health information has value only to the extent that it is clearly delivered, understood, accepted, and remembered by members of its intended audience [14,15]. Even when these conditions are met, health information influences subsequent health actions only to the extent that recipients are motivated to comply, have a strong belief in their ability to perform the recommended behaviors (i.e., high self-efficacy), and have the skills necessary to perform the behavior in real-world contexts [16,17]. To be effective, behavioral RMPs must ensure that all of these conditions are met, or they must have means to enforce the performance of the behavior.

### First-Generation Approach to Risk Management: 1982–1987

Accutane, the first approved form of isotretinoin, was brought to market by Hoffman-La Roche (Roche) in September 1982. Anticipating teratogenic effects based on animal research, Roche distributed the drug with a warning on its Food and Drug Administration (FDA)–approved professional package insert and a patient brochure which warned against pregnancy.

By October 1983, Roche had received seven reports of Accutane-caused fetal malformations. Roche took steps to heighten the strength of the pregnancy warning: language about the drug's teratogenic potential was placed in bold on the package insert; warning letters were sent to prescribers; and red warning stickers were sent to pharmacists and wholesalers to be placed on the drug bottles [18].

### Second-Generation Approach to Risk Management: 1988–2001

Reports of fetal malformations continued, leading the FDA in 1988 to call for a stronger RMP [19]. As a result, Roche developed the Pregnancy Prevention Program (PPP). PPP was a comprehensive educational program which included materials for the prescriber to use in counseling the female patient about the importance of avoiding pregnancy while taking Accutane. Changes in the labeling and packaging included the addition of an icon intended to communicate the need to avoid pregnancy and a “black box” warning on the package insert [20]. More significantly, the program required that prescribing physicians make three risk management procedures mandatory for all female patients as a precondition for prescribing: the signing of a patient consent form, the taking of a pregnancy test seven days prior to beginning treatment, and the selection of two reliable forms of birth control (unless patients indicated that they were abstinent). While these procedures were labeled as “requirements” for prescribing Accutane on the package insert, no mechanism was put in place to enforce that they were carried out. To facilitate the program's implementation, Roche provided prescribing physicians with forms and educational materials to use in counseling patients.

A Roche-sponsored evaluation of the PPP program, which was based on a voluntary sample of female Accutane patients, found that 99% of patients recalled having been told to avoid pregnancy [7]. However, 36% of patients failed to receive a pregnancy test prior to starting therapy, and 15% did not recall being told to use birth control for one month prior to starting therapy [7]. The pregnancy rate was found to be 3.4 pregnancies per 1,000 courses of isotretinoin [7]. A later study found the pregnancy rate had been reduced to 2.8 per 1,000, still resulting in scores of exposed pregnancies each year [19]. In a separate study of 14 Accutane-exposed pregnancies, eight women were found to have used no contraception at all, and another five used only one method, when two were recommended [21]. In evaluating the program's success, the FDA concluded that the voluntary and information-based RMP was not sufficiently effective in influencing the contraceptive behavior of female patients and in reducing the number of exposed pregnancies [22].

### Third-Generation Approach to Risk Management: 2002–2005

In 2002, the FDA called for further revisions to the risk management program for Accutane and other more recently approved generic versions [22]. The FDA mandated that a medication guide —a written drug information sheet—be given to patients by pharmacists along with all new and refill prescriptions of isotretinoin. Building on its PPP, Roche created SMART (System to Manage Accutane Related Teratogenicity), which upgraded the risk management practices to include improved education for female patients and two negative pregnancy tests. It also included a novel risk management tool: the required use of a yellow qualification sticker on all prescriptions issued. The qualification stickers—which were to be filled out by prescribers and placed on prescriptions—were intended to serve as a reminder to prescribers of their responsibilities in insuring that female patients were qualified for isotretinoin (i.e., that they had been given two pregnancy tests and found not to be pregnant, and that they had chosen two forms of birth control). While prescribers were sent the qualification stickers only after signing a letter which detailed qualification requirements, no enforcement mechanism was put in place to insure that prescribers gave qualification stickers only to patients who were truly eligible. Pharmacists were required to fill only those Accutane prescriptions with an affixed sticker [22]. All of the generic manufacturers fielded similar risk management programs.

An evaluation of the SMART program found that 98% of women taking Accutane knew to avoid pregnancy [23] and 92% recalled having received a prescription with a qualification sticker [22]. However, of women at risk for pregnancy, 34% failed to receive the required two pregnancy tests [22]. Of women who were at risk for pregnancy and non-abstinent, 54% did not use two forms of birth control [22]. More importantly, the pregnancy rate did not decline substantially below that achieved under the PPP [22]. Thus, while the inclusion of a reminder system—the use of the yellow qualification stickers—did increase the proportion of women who took pregnancy tests and used appropriate birth control [22], these changes fell short of expectations and did not lead to a sufficient reduction in the number of exposed pregnancies. Although the evaluations of this RMP were not able to pinpoint the reasons for shortcomings in the system performance, prescriber under-compliance with qualification requirements and patients' lack of motivation, selfefficacy, and/or ability to use two forms of contraception throughout the course of treatment were likely contributors.

### Fourth-Generation Approach to Risk Management: 2005–Current

SMART's limited success in further reducing isotretinoin-exposed pregnancies led the FDA to request that Roche and other isotretinoin manufacturers create a more rigorous performance-linked RMP. Stringent performance-linked programs have been used for a handful of drugs including thalidomide and clozapine, though none of these are as widely prescribed as isotretinoin. In response to the request, all four manufacturers collaborated to create the iPLEDGE program.

iPLEDGE requires that all parties in the distribution chain (i.e., health-care professionals who prescribe isotretinoin, pharmacies that dispense isotretinoin, and wholesalers that distribute isotretinoin) and all patients receiving prescriptions—male and female—register in a single database online (https://www.ipledgeprogram.com) or by telephone. Prior to dispensing to females, providers must enter the results of two negative pregnancy tests, and specify two forms of contraception that the patient agrees to use, and must on a monthly basis administer and verify the negative results of pregnancy tests, provide additional pregnancy prevention counseling, and re-identify the patient's contraceptive choices. Prior to receiving their medication, patients must register with the system, sign a consent form, and identify their two forms of birth control. On a monthly basis, they must indicate their two forms of contraception (which must match the provider's entry) and must answer questions which demonstrate their knowledge of the teratogenic effects of isotretinoin. Pharmacists are permitted to dispense only if the web-based system shows that the patient and provider have complied with all requirements of the system.

iPLEDGE represents the most rigorous risk management program in history for such a widely prescribed drug.

As the program is relatively new, there has been no formal evaluation of iPLEDGE to date. According to FDA reports, the program appears to have been widely adopted. Between December 30, 2005 and March 23, 2006, 22,000 prescribers and 71,700 patients registered with iPLEDGE [13]. However, based on news reports, the program has not been well received, especially by dermatologists, the primary prescribers of isotretinoin [24]. Dermatologists have complained that the new program is inflexible and confusing, and has too many requirements. Many were frustrated initially by technical glitches from the Web-based system coupled with inadequate telephone support [24]. There have also been reports of patients having difficulty in complying with the system's numerous requirements [25].

### The Advantages of iPLEDGE

iPLEDGE has the potential to improve upon the previous RMPs by identifying existing pregnancies before treatment has begun, by detecting new, unplanned pregnancies earlier, and by increasing patients' knowledge and motivation about the importance of complying with program requirements. Under iPLEDGE, prescribers must ensure two negative pregnancy tests before a patient can start treatment. This represents a significant improvement because under SMART, over one-third of women did not receive these tests [22], and an estimated 13% of exposed pregnancies existed prior to the start of treatment [26]. Furthermore, requiring monthly pregnancy tests, pledging in advance to use two forms of birth control, and the myriad monthly reminders received by both patients and prescribers when they log onto the iPLEDGE system should have a beneficial effect on a patient's motivation to comply.

Effective use of contraception requires knowledge, motivation, skill, and for some methods, a high degree of self-efficacy [27]. Under SMART, the most commonly reported reasons for pregnancy during isotretinoin therapy were unsuccessful attempts at abstinence, use of ineffective contraception, inconsistent use of contraception, unexpected sexual activity, and contraceptive failure [20]. It remains an open question the degree to which iPLEDGE will result in better pregnancy prevention skills, higher levels of self-efficacy about pregnancy prevention and, ultimately, pregnancies prevented.

### Concerns about iPLEDGE

Despite the advantages of iPLEDGE, we have several concerns about the new program. Some patients who have insufficient skills in using contraceptives will be in the care of providers (in most cases dermatologists) who may not have time or sufficient knowledge to counsel their patients about contraceptive use. Some patients, especially teenagers, who represent a significant proportion of isotretinoin users, will be unwilling to communicate openly with their health-care provider about their sexual activity [22]. Some patients are likely to be insufficiently motivated to follow through with their plans to use two forms of contraception for every sexual episode over a five to six month course of treatment. Furthermore, as isotretinoin remains widely available over the Internet without a prescription [28], many people will obtain isotretinoin outside of the iPLEDGE RMP. This problem may increase as people try to avoid the stringent requirements that are part of iPLEDGE.

Also of concern is that the iPLEDGE program may have been suboptimally designed, and as a result, may place an unnecessary burden on patients and providers. Core principles of program design and development dictate that: (1) programs should be developed with much input from the end-users of the systems—in this case prescribers, patients, pharmacies, and wholesalers; (2) programs should receive extensive user testing before being released to the public; and (3) programs should be developed and maintained within a context of total quality management [29]. These basic principles appear to have been somewhat violated in the development of iPLEDGE.

Prior to the program's release, limited input was solicited from the iPLEDGE Scientific Advisory Board, a group formed in early 2005 to represent the various stakeholder groups. The program became mandatory before it was sufficiently tested, and users who were required to use the system were initially faced with navigating through technical problems and computer glitches [24]. It remains to be seen the degree to which principles of total quality management—including a strong focus on process measurement and continuous improvements—will be embraced.

### Ways to Improve iPLEDGE

We see several opportunities to improve iPLEDGE to address some of these concerns. The fact that iPLEDGE is managed primarily via a Web site presents excellent opportunities for tailored and multimedia patient education that currently have not been embraced. As designed, the Web site's primary function is data entry. The site currently contains a minimal amount of patient education material, and the information present is exclusively text-based, presented in 9 point font, and written at a 12th grade reading level, which is substantially higher than the ability of the average adult [30].

We recommend the addition of appropriate multimedia educational content to: (1) reinforce important information (i.e., the risks associated with isotretinoin and the need for two forms of contraception), and perhaps more importantly, (2) provide an effective means of conveying difficult or sensitive information that is not otherwise being effectively communicated (e.g., information on various contraceptive methods and how to appropriately use them, complete with animation/video to model various techniques). Furthermore, we recommend audience testing the Web site with all categories of users—current isotretinoin patients, prescribers, and pharmacists—to ensure that the content of the Web site is clearly communicated, easy to use, and meaningful. It may also be necessary to simplify the program requirements so that there is less burden on patients and providers, and less incentive for patients to circumvent program procedures.

The required monthly knowledge-check questions in iPLEDGE—intended to determine if the patient understands the need for two forms of birth control and the potential of isotretinoin to cause birth defects—is a novel approach to encouraging patient compliance. However, given that nearly all patients said they understood they were not supposed to get pregnant under the previous RMPs [8,22,23], a more appropriate focus for these questions is to ensure that patients have actionable knowledge about pregnancy prevention and are properly using the contraceptive methods that they selected. Incorrect responses should elicit presentation of appropriate educational material.

By adding an e-mail function, iPLEDGE could automatically generate and send tailored reminders and other relevant information to patients. (For phone users, this information could be available via regular mail). Such proactive contacts might be especially valuable in reminding patients of the need for pregnancy prevention during the one month interval after therapy ends when many patients will lose contact with iPLEDGE. Under SMART, 32% of Accutane-exposed pregnancies occurred during this post-therapy period [26].

We believe an important general lesson from evaluations of isotretinoin RMPs is that RMPs need to be designed with input from users in all phases of a program's development. Furthermore, information alone may not be sufficient to manage behavior-based medical risks, especially when mitigating those risks requires that the behaviors be performed consistently, and with considerable motivation and skill, over long periods of time. Having program “requirements” in the absence of enforcement mechanisms for those requirements may not be sufficient to manage these types of medical risks. Finally, information and performance-linked approaches should not been seen as either/or alternatives, but complements in achieving sustained behavior change.

### The Case for Performance-Linked Systems

While performance-linked systems undoubtedly place a burden on everyone in the system, from a risk management perspective, there is a compelling case to be made for them. For example, Directly-Observed Treatment Short-Course (DOTS) has become the internationally recommended tuberculosis control strategy despite the demands it places on patients and the health-care system. The performance of iPLEDGE will likely influence a dialogue about whether the demands it places on patients and the health-care system are tolerable, and whether performance-linked systems should be extended to other medications when the potential negative consequences to patients, their offspring, or society at large are severe.

### Acknowledgments

The authors would like to thank Dr. Howard Maibach and Dr. Jonathan Wilkin for providing comments on the manuscript, Dr. Jill Lindstrom for help in understanding the regulatory history of isotretinoin, and Ms. Jenna Williams-Bader for research assistance.

## Steven R. Feldman's Viewpoint: A Balanced Approach May Be The Best Way To Reduce Isotretinoin Birth Defects

Acne can disfigure patients physically and emotionally, while disabling them socially. This inflammatory disease can have a dramatic impact on patients' lives [31]. In the not-too-distant past, there was little dermatologists could do for patients with the most severe forms. Now, patients (and their parents) do not need to suffer the physical and emotional scars of acne. For even the most severely affected patients, the disease is treatable, even curable, with isotretinoin.

Unfortunately, isotretinoin is not without side effects. Patients must be warned about the rare risk of severe depression. While I am convinced this rare side effect is real, isotretinoin probably prevents more depression in teens than it causes [32,33]. More important is the risk of birth defects occurring when a fetus is exposed to isotretinoin. Isotretinoin is a powerful teratogen used in the treatment of a disease that occurs in young women of child-bearing potential. Physicians, and these patients, bear a heavy responsibility when using isotretinoin.

The benefits accrued to society from using isotretinoin outweigh the risks.

Under guidance from the FDA, isotretinoin manufacturers have instituted increasingly stringent programs to prevent isotretinoin-exposed pregnancies. Whether any of these programs have substantially reduced isotretinoin-exposed pregnancies beyond the efforts already taken by physicians in the care of their patients is not known [34]. It is difficult for me to believe that any one-size-fits-all program could be more effective in preventing pregnancy than the careful efforts of a physician working with their patient. But when it comes to preventing birth defects, I'm glad for any help I can get.

Unfortunately, we don't know whether even the most stringent pregnancy prevention program so far, iPLEDGE, reduces the rate of exposed pregnancies. The pregnancy prevention programs have been based largely on monthly pregnancy testing. The history of the isotretinoin pregnancy prevention programs and a recent retrospective study show that pregnancy testing doesn't prevent pregnancy [35]. I sometimes wonder if doing this testing gives some patients a cavalier attitude based on the false impression that catching pregnancies early will prevent isotretinoin-induced birth defects. Catching an exposed pregnancy early does not prevent isotretinoin teratogenicity. It is essential to make sure patients don't get pregnant in the first place.

One strength of iPLEDGE is accountability. At the very least, iPLEDGE will help prevent the 13% of exposed pregnancies that occur when someone who was already pregnant takes the drug. But iPLEDGE also has many weaknesses. The program was implemented without any testing to determine whether it actually reduces pregnancy risk. Beyond that, the hassles of the program leave less time to focus on the single most important message: you must not become pregnant while isotretinoin is in your body.

The apparently untested recommendations for improving iPLEDGE described by Abroms et al. may help, but they may not. A lesson from the history of past programs is that new programs should be tested before implementation. Until then, physicians must continue to use isotretinoin with great caution. Isotretinoin should be a last-line acne treatment for women of child-bearing potential. Typically, these women should first be treated with a combination of topical antibiotics and retinoids [36], oral antibiotics (probably a second one if the first doesn't work), and birth control pills for several months before using isotretinoin. Birth control pills are an effective, FDA-approved treatment for acne in women. By using them first, some isotretinoin use might be averted altogether. If isotretinoin is still necessary, the woman will already have been on birth control pills for several months and will have had time for education about the drug and proper (and, we all hope, effective) pregnancy prevention counseling.

When treating a patient with severe acne unresponsive to other treatments, having access to isotretinoin is a godsend. Perhaps some people believe that even one isotretinoin-induced birth defect is too many and think that isotretinoin use should be stopped altogether. Stopping birth defects is an emotionally powerful goal. Yet dermatologists often see patients with horrific scars from acne, patients whose suffering could have been prevented by early treatment with isotretinoin.

There are both risks and benefits to isotretinoin. There has been some effort to quantify and compare these risks and benefits [37]. Despite patients' and physicians' efforts at pregnancy prevention, there are still a limited number of fetal isotretinoin exposures. Yet because acne is so horrific and so common, even the most conservative risk/benefit analysis finds that, overall, isotretinoin provides far more benefit than risk. Even if it were used for milder forms of acne, the benefits accrued to society from using isotretinoin outweigh the risks in quantitative analyses. While everyone (patients, their families, physicians, the media, interest groups, and politicians) would like to see the risk of isotretinoin-induced birth defects reduced to the lowest possible level, those who see the benefits of isotretinoin do not want patients to suffer (and suffer greatly) by losing isotretinoin entirely.

## Abbreviations

PPP: Pregnancy Prevention Program
RMP: risk management program
SMART: System to Manage Accutane Related Teratogenicity

## References

[pmed-0030483-b001] Lehmann HP, Robinson KA, Andrews JS, Holloway V, Goodman SN (2002). Acne therapy: A methodologic review. J Am Acad Dermatol.

[pmed-0030483-b002] Jewett S, Ryan T (1985). Skin disease and handicap: An analysis of the impact of skin conditions. Soc Sci Med.

[pmed-0030483-b003] Cunliffe WJ (1986). Acne and unemployment. Br J Dermatol.

[pmed-0030483-b004] Cotterill JA, Cunliffe WJ (1997). Suicide in dermatological patients. Br J Dermatol.

[pmed-0030483-b005] Strauss JS, Rapini RP, Shalita AR, Konecky E, Pochi PE (1984). Isotretinoin therapy for acne: Results of a multicenter dose-response study. J Am Acad Dermatol.

[pmed-0030483-b006] Lammer EJ, Chen DT, Hoar RM, Agnish ND, Benke PJ (1985). Retinoic acid embryopathy. N Engl J Med.

[pmed-0030483-b007] Mitchell AA, Van Bennekom CM, Louik C (1995). A pregnancy-prevention program in women of childbearing age receiving isotretinoin. N Engl J Med.

[pmed-0030483-b008] Lindstrom J (2004). Isotretinoin: Background and regulatory history. FDA Joint Dermatologic and Ophthalmic Drugs & Drug Safety and Risk Management Advisory Committee Meeting. http://www.fda.gov/ohrms/dockets/ac/04/slides/4017s1.htm.

[pmed-0030483-b009] Wysowski DK, Pitts M, Beitz J (2001). An analysis of reports of depression and suicide in patients treated with isotretinoin. J Am Acad Dermatol.

[pmed-0030483-b010] Stern RS (2000). Medication and medical service utilization for acne 1995–1998. J Am Acad Dermatol.

[pmed-0030483-b011] Chen K, White TJ, Juzba M, Chang E (2002). Oral isotretinoin: An analysis of its utilization in a managed care organization. J Manag Care Pharm.

[pmed-0030483-b012] Wysowski DK, Swann J, Vega A (2002). Use of isotretinoin (Accutane) in the United States: Rapid increase from 1992 through 2000. J Am Acad Dermatol.

[pmed-0030483-b013] Food and Drug Administration (2006). iPLEDGE update. http://www.fda.gov/cder/drug/infopage/accutane/iPLEDGEupdate200603.htm.

[pmed-0030483-b014] McGuire WJ (1984). Public communication as a strategy for inducing health-promoting behavioral change. Prev Med.

[pmed-0030483-b015] Synder LB, Hamilton MA, Mithchell EW, Kiwanuka-Tondo J, Fleming-Milici F (2004). A meta-analysis of the effect of mediated health communication campaigns on behavior change in the United States. J Health Commun.

[pmed-0030483-b016] Bandura A (1986). Social foundations of thought and action: A social cognitive theory.

[pmed-0030483-b017] Glanz K, Rimer BK, Lewis FM (2002). Health behavior and health education: Theory, research and practice.

[pmed-0030483-b018] Food and Drug Administration (1983). http://www.fda.gov/ohrms/dockets/ac/accutane.htm.

[pmed-0030483-b019] Food and Drug Administration (1988). Dermatologic Drugs Advisory Committee Transcript: April 26, 1988. http://www.fda.gov/ohrms/dockets/ac/accutane.htm.

[pmed-0030483-b020] Perlman SE, Leach EE, Dominguez L, Ruszkowski AM, Rudy SJ (2001). “Be smart, be safe, be sure”. The revised Pregnancy Prevention Program for women on isotretinoin. J Reprod Med.

[pmed-0030483-b021] Honein MA, Paulozzi LJ, Erickson JD (2001). Continued occurrence of Accutane-exposed pregnancies. Teratology.

[pmed-0030483-b022] Brinker A, Kornegay C, Nourjah P (2005). Trends in adherence to a revised risk management program designed to decrease or eliminate isotretinoin-exposed pregnancies. Arch Dermatol.

[pmed-0030483-b023] Shiff SA (2004). Overview of SMART Program. FDA Joint Dermatologic and Ophthalmic Drugs & Drug Safety and Risk Management Advisory Committee Meeting. http://www.fda.gov/ohrms/dockets/ac/04/slides/4017s1.htm.

[pmed-0030483-b024] Darves B (2006 March 9). Dermatologists frustrated with problematic iPledge program. Medscape Medical News. http://www.medscape.com/viewarticle/525664.

[pmed-0030483-b025] Harris G (2006 February 11). System said to fail to steer women from acne drug. The New York Times.

[pmed-0030483-b026] Huber MH (2004). Review of Data from SMART Program. FDA Joint Dermatologic and Ophthalmic Drugs & Drug Safety and Risk Management Advisory Committee Meeting. http://www.fda.gov/ohrms/dockets/ac/04/slides/4017s1.htm.

[pmed-0030483-b027] Brown SS, Eisenberg L (1995). The best intentions: Unintended pregnancy and the well-being of children and families.

[pmed-0030483-b028] Honein MA, Moore CA, Erickson JD (2004). Can we ensure the safe use of known human teratogens?. Drug Saf.

[pmed-0030483-b029] Andreasen AR (1995). Marketing social change: Changing behavior to promote health, social development, and the environment.

[pmed-0030483-b030] Doak CC, Doak LG, Root JH (1996). Teaching patients with low literacy skills.

[pmed-0030483-b031] Chen SC, Bayoumi AM, Soon SL, Aftergut K, Cruz P (2004). A catalog of dermatology utilities: A measure of the burden of skin diseases. J Investig Dermatol Symp Proc.

[pmed-0030483-b032] Rohrback JM, Fleischer AB, Krowchuk DP, Feldman SR (2004). Depression is not common in isotretinoin-treated acne patients. J Dermatolog Treat.

[pmed-0030483-b033] Jick SS, Kremers HM, Vasilakis-Scaramozza C (2000). Isotretinoin use and risk of depression, psychotic symptoms, suicide, and attempted suicide. Arch Dermatol.

[pmed-0030483-b034] Fleischer AB, Simpson JK, McMichael A, Feldman SR (2003). Are there racial and sex differences in the use of oral isotretinoin for acne management in the United States?. J Am Acad Dermatol.

[pmed-0030483-b035] Cheetham TC, Wagner RA, Chiu G, Day JM, Yoshinaga MA (2006). A risk management program aimed at preventing fetal exposure to isotretinoin: Retrospective cohort study. J Am Acad Dermatol.

[pmed-0030483-b036] Thevarajah S, Balkrishnan R, Camacho FT, Feldman SR, Fleischer AB (2005). Trends in prescription of acne medication in the US: Shift from antibiotic to non-antibiotic treatment. J Dermatolog Treat.

[pmed-0030483-b037] Jordan AY, Parks L, Chen SC, Higgins K, Fleischer AB (2005). Does the teratogenicity of isotretinoin outweigh its benefits?. J Dermatolog Treat.

